# Connective Tissue Growth Factor Overexpression in Cardiomyocytes Promotes Cardiac Hypertrophy and Protection against Pressure Overload

**DOI:** 10.1371/journal.pone.0006743

**Published:** 2009-08-25

**Authors:** Anna N. Panek, Maximilian G. Posch, Natalia Alenina, Santhosh K. Ghadge, Bettina Erdmann, Elena Popova, Andreas Perrot, Christian Geier, Rainer Dietz Ingo Morano, Michael Bader, Cemil Özcelik

**Affiliations:** 1 Department of Cardiovascular and Metabolic Disease Research, Max Delbrück Center for Molecular Medicine, Berlin, Germany; 2 Experimental and Clinical Research Center (ECRC) at the Max Delbrück Center for Molecular Medicine, Berlin, Germany; 3 Department of Cardiology, Charité-Universitätsmedizin, Campus Virchow Klinikum, Berlin, Germany; University Hospital Vall d'Hebron, Spain

## Abstract

Connective tissue growth factor (CTGF) is a secreted protein that is strongly induced in human and experimental heart failure. CTGF is said to be profibrotic; however, the precise function of CTGF is unclear. We generated transgenic mice and rats with cardiomyocyte-specific CTGF overexpression (CTGF-TG). To investigate CTGF as a fibrosis inducer, we performed morphological and gene expression analyses of CTGF-TG mice and rat hearts under basal conditions and after stimulation with angiotensin II (Ang II) or isoproterenol, respectively. Surprisingly, cardiac tissues of both models did not show increased fibrosis or enhanced gene expression of fibrotic markers. In contrast to controls, Ang II treated CTGF-TG mice displayed preserved cardiac function. However, CTGF-TG mice developed age-dependent cardiac dysfunction at the age of 7 months. CTGF related heart failure was associated with Akt and JNK activation, but not with the induction of natriuretic peptides. Furthermore, cardiomyocytes from CTGF-TG mice showed unaffected cellular contractility and an increased Ca^2+^ reuptake from sarcoplasmatic reticulum. In an ischemia/reperfusion model CTGF-TG hearts did not differ from controls.

Our data suggest that CTGF itself does not induce cardiac fibrosis. Moreover, it is involved in hypertrophy induction and cellular remodeling depending on the cardiac stress stimulus. Our new transgenic animals are valuable models for reconsideration of CTGF's profibrotic function in the heart.

## Introduction

Heart failure is an increasing health problem worldwide [1]. The mortality rate averages 30% within the first year after diagnosis irrespective of treatment [1], [2]. Heart failure has numerous causes; however, the major pathomechanisms are universal [3]. The cardiac remodeling cascade involves initiation of adaptive cellular hypertrophy followed by left ventricular dilatation, decreased systolic function, cardiomyocyte loss, and development of fibrosis. The profibrotic and prohypertrophic changes in the heart are mainly driven by transforming growth factor-β (TGF-β) [4], [5], [6].Connective tissue growth factor (CTGF; also known as CCN2) is an extracellular matrix-secreted protein that is induced by TGF-β [7], [8]. The protein belongs to the highly conserved CCN family (Cyr61, CTGF, and NOV) of growth factors that exerts a wide range of biological functions [9], [10]. CTGF regulates such diverse cellular processes as extra-cellular matrix (ECM) deposition, wound repair, angiogenesis, migration, and differentiation, as well as cell survival and proliferation [9]. However, the most prominent feature of CTGF is its overexpression in fibrosis of various organs including lung, kidney, skin, and heart [11], [12], [13], [14], [15], [16], [17], [18], [19]. Angiotensin II (Ang II), epinephrine, and mechanical stress induce CTGF in cultured cardiomyocytes [18], [20]. Other studies showed that CTGF also exhibited prohypertrophic properties on cardiomyocytes [21]. Furthermore, serum CTGF has been proposed as a heart failure biomarker [19], [22]. The diversity of findings regarding CTGF and its role in heart failure prompted us to investigate CTGF function in two different rodent models of CTGF overexpression in the heart.

## Results

### Expression and localization of CTGF in CTGF-TG animals

**Figure 1:**
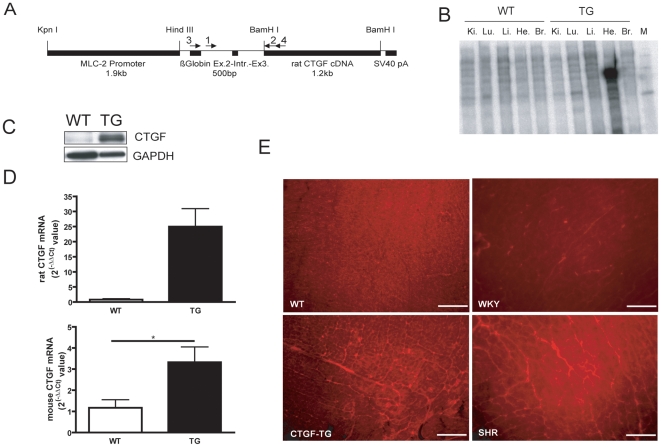
Construction of cardiomyocyte-specific CTGF-TG mice. (A) Schematic structure of the transgene. 1 and 2 indicate primers used for genotyping and 3 and 4 show primers used to generate the probe for CTGF ribonuclease protection assay. MLC-2 (myosin light chain-2) promoter; rabbit β-globin exon 2 and 3 for enhanced expression of the transgene; SV40 pA, polyadenylation signal from Simian virus 40. (B) Expression of CTGF mRNA in hearts (He) and other organs (kidney, lung, liver, brain) of transgenic mice shown by ribonuclease protection assay. (C) Western Blot analyses of CTGF protein overexpression in CTGF-TG mouse hearts. GAPDH protein expression was used as loading control. (D) Quantification of the transgenic and endogenous CTGF mRNA expression by TaqMan PCR. (WT n = 4, TG n = 4; *, P<0.05 (E) Histological staining of CTGF in WT and CTGF-TG mice as well as in Wistar-Kyoto rats (WKY) as control for spontaneously hypertensive rat (SHR). Scale bars designate a length of 100 µm.

We constructed a transgene using the mouse MLC-2 promoter, rabbit β-globin exon 2, intron 2 and exon 3, rat CTGF cDNA and a polyA signal SV40-virus (Figure 1A). We obtained a transgenic mouse and a transgenic rat line, which showed an abundant expression of CTGF mRNA and protein in the heart but not in other tissues (Figure 1B, C and supplemental Figure S1A, S1B). The overexpression of rat CTGF cDNA in the mouse allowed us to distinguish between endogenous CTGF expression and CTGF mRNA derived from the transgene. As shown by quantitative Taqman-PCR with specific CTGF mouse and rat primers and probes, the endogenous CTGF expression was strongly induced in CTGF-TG mouse hearts (Figure 1D) indicating an autocrine mechanism driving the CTGF expression in the heart. As shown by immunohistochemistry CTGF was marginally expressed in WT hearts (Figure 1E) while in the transgenic heart ventricles CTGF was abundantly present in the interstitial space (Figure 1E). No CTGF specific staining signal was observed in non-hypertensive WKY-control rats (Figure 1E) or in control staining performed without primary anti-CTGF-antibody (data not shown). In comparison, hearts from 6-months-old spontaneously hypertensive rats (SHR) displayed a pronounced hypertrophy and fibrosis accompanied by high CTGF upregulation with a similar localization in the interstitial space (data not shown).

### Age-dependent heart failure in CTGF-TG mice

**Figure 2:**
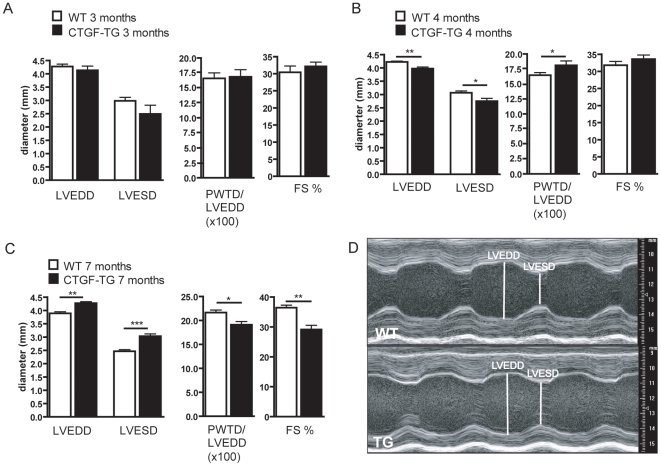
Characterization of CTGF-TG mice of different ages by echocardiography. (A, B, C) Measurements of LVEDD, LVESD, calculated ratio of PWD/LVEDD and FS of 3-months-old mice (A, WT n = 9 TG n = 7), 4-months-old mice (B, WT n = 10, TG n = 9) and 7-months-old mice (C, WT n = 7, TG n = 6). * P<0.05; ** P<0.01; *** P<0.001. (D) Examples of M-mode echocardiography in CTGF-TG and WT mice at age of 7 months.

CTGF-TG mice and rats developed normally and had no immediate increased mortality. To investigate the role of CTGF in cardiac performance we examined mice by echocardiography over time (Figure 2D). At 3 months of age, the average LVEDD and LVESD as well as FS were similar in control and CTGF-TG mice (Figure 2A). Compensatory hypertrophy, dilatation, and finally loss of contractility became apparent at a later age. At age of 4 months, the LVEDD reached an average value of 3.9 mm in transgenic mice, compared to 4.2 mm in normal littermates (p<0.01). Similarly, a reduction of LVESD and an increase of PWTD, both significant, were observed indicating a hypertrophic change in the left heart chamber architecture. These changes were associated with a slight but not significant increase in FS (Figure 2B). Hypertrophy was followed by ventricular dilatation at the age of 7 months: the CTGF-TG mice developed remarkably increased ventricular diameters (Figure 2C) and a severe loss of the cardiac function compared to WT animals (Figure 2C, 2D). Thus, CTGF overexpression in cardiomyocytes caused age-dependent heart dysfunction with a disease course going from compensatory hypertrophy to ventricular dilatation and systolic heart failure.

### CTGF does not induce cardiac fibrosis in murine or rat hearts

**Figure 3:**
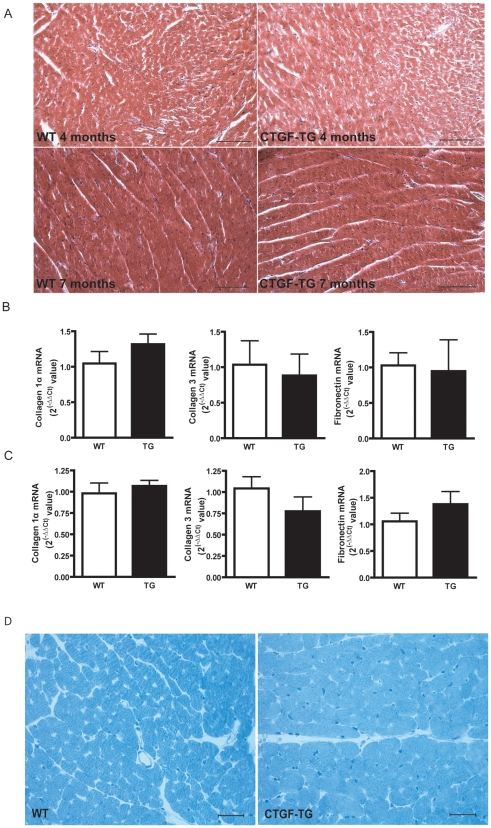
Assessment of cardiac fibrosis at age of 4 and 7 months in CTGF-TG and WT mice. (A) Masson's trichrome stained cardiac sections of WT and CTGF-TG mice at age of 4 and 7 months. Scale bars designate a length of 100 µm. (B, C) Quantification of the collagen 1α, collagen 3 and fibronectin mRNA expression by TaqMan-PCR performed at age of 4- (B) and 7 months (C) (n = 4 per group). (D) High magnifications of toluidine blue-stained semithin sections, with myofiber hypertrophy apparent in 7 months old CTGF-TG hearts. Scale bars designate a length of 20 µm.

Since a large body of evidence implicates CTGF as a profibrotic factor, we addressed the question whether CTGF overexpression would induce cardiac fibrosis. In CTGF-TG mice and rats no fibrotic changes were detectable as assessed by Masson's trichrome and Sirius red stainings (Figure 3A and supplemental Figure S1C and S1E). Accordingly, the mRNA levels of collagen 1α, collagen 3, and fibronectin were not increased in CTGF-TG mice and rats (Figure 3B, 3C and supplemental Figure S1D). Yet, an increased diameter of murine CTGF-TG cardiomyocytes could be detected by tissue structural analyses in hearts from 7 months old mice (Figure 3D). Taken together, overexpression of CTGF in cardiomyocytes did not induce fibrosis neither in murine nor in rat hearts, but was rather associated with hypertrophic changes at the cellular level.

### CTGF overexpression is associated with Akt- and JNK-activation

**Figure 4:**
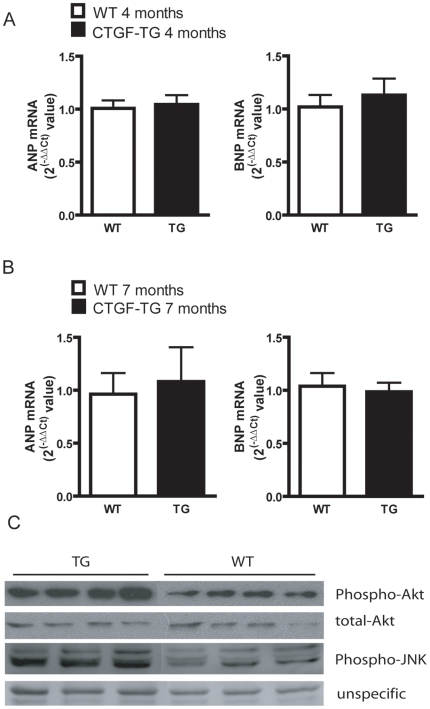
Quantification of expression and activation of cardiac hypertrophy markers. (A, B) Quantification of ANP and BNP mRNA expression by TaqMan-PCR in 4- (A) and 7 months (B) old mice (n = 4 per group). (C) Quantitative analysis of the phosphorylation status of Akt and JNK at age of 7 months. The band intensities of Akt- or JNK-phosphoproteins are normalized with those of unphosphorylated Akt or unspecific bands respectively.

To elucidate the cellular mechanisms underlying the observed phenotype in CTGF-TG mice, we determined the mRNA expression levels of two hypertrophy markers, atrial natriuretic peptide (ANP) and brain natriuretic peptide (BNP). TaqMan real time PCR showed no changes in the ANP and BNP expression between WT and transgenic mice at 4 and 7 months of age (Figure 4A and 4B). To further examine whether CTGF initiates hypertrophic signals immunoblot analyses were performed on lysates from 7-month-old murine hearts with phospho-specific antibodies. As shown in Figure 4C CTGF-TG hearts displayed an activation of the Akt and JNK pathways. These data suggest that the cardiac changes in transgenic mice were associated with the activation of the Akt and JNK pathways but were independent of ANP and BNP expression.

### CTGF overexpression preserves the cardiac function under Ang II-induced pressure overload conditions

**Figure 5:**
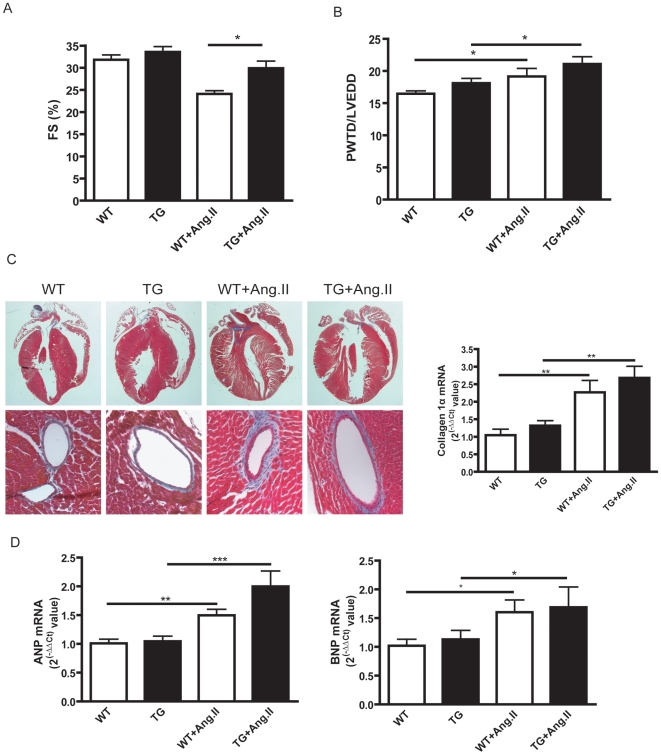
Characterisation of cardiac function, fibrosis and hypertrophy under pressure overload conditions (3.5 months of age). (A, B) Echocardiographic measurements of fractional shortening (A) and interventricular septum diameter in diastole (B) in WT (n = 10) and CTGF-TG (n = 11) mice treated with Ang II (*p<0.05; ** p<0.01, *** p<0.001) (C) Masson's trichrome stained cardiac sections of mice treated with Ang II and quantification of collagen 1α mRNA expression by Taqman-PCR. (D) Quantification of ANP and BNP mRNA expression by TaqMan-PCR in mice treated with Ang II (n = 4 per group).

In WT mice, echocardiography revealed a significant reduction in systolic function after Ang II infusion, as indicated by decreased FS (Figure 5A). This reduction was almost completely abrogated in treated CTGF-TG animals (Figure 5A). In CTGF-TG mice, the contractile performance after Ang II was preserved to the level of mice receiving only a sham infusion. As expected after Ang II infusion, the ratio PWTD/LVEDD was increased (Figure 5B). However, these increases were not different between WT and CTGF-TG mice. The subsequent stainings showed an increased degree of fibrosis, which was not different between CTGF-TG mice and controls (Figure 5C). Accordingly, collagen 1α mRNA was elevated in WT and transgenic animals after treatment with Ang II, but both groups were similarly affected (Figure 5C). The mRNA quantification of the hypertrophy markers ANP and BNP showed a significant increase after Ang II infusion (Figure 5D). However, there were no significant differences between CTGF-TG and WT mice. Altogether, these data suggest that CTGF overexpression has a beneficial effect on cardiac function in an acute pressure-overload heart failure model. This protection is not due to elevated levels of ANP and BNP. Moreover, the CTGF overexpression does not affect the development of cardiac fibrosis under hypertrophic conditions.

### Improved Ca^2+^ handling in CTGF TG cardiomyocytes

To further elucidate the cellular mechanisms in CTGF-TG hearts at a single cell level, we characterized intracellular Ca^2+^ transients of isolated cardiomyocytes. The recordings of contraction kinetics of single cardiomyocytes at 3 months of age revealed that the basal cell length and contractile function were similar in transgenic and WT cardiomyocytes (supplemental Figure S2). In both groups, we observed a comparable FS as well as similar contraction and relaxation rates (supplemental Figure S2A-S2D). In order to record the intracellular Ca^2+^-transient, we loaded cardiomyocytes with Ca^2+^-sensitive Fura-2 dye and observed a slightly, but robustly reduced cytosolic Ca^2+^-content in diastole in transgenic cardiomyocytes compared to WT cells (supplemental Figure S2E-S2H). These results indicate an improved diastolic clearance of Ca^2+^ from the cytosol in CTGF-TG cardiomyocytes. Further, we assayed the Ca^2+^ transient amplitude and the velocity of Ca^2+^ influx and -efflux from sarcoplasmatic reticulum. Yet, the last parameters were not found to be changed in transgenic cardiomyocytes. Taken together, overexpression of CTGF in cardiomyocytes is accompanied by an enhanced Ca^2+^ clearance from the cytosol. However, at this time point the modification of the Ca^2+^ cycling is not reflected in an altered cellular contractility.

### CTGF-overexpression and acute ischemia

Several studies reported an upregulation of CTGF upon ischemic conditions.[23], [24] In order to examine the effects of CTGF overexpression on the ischemic heart disease, we subjected isolated CTGF-TG and WT mouse hearts to 40 minutes ischemia with subsequent 60 minutes reperfusion phase. In the setting of the working heart model, we monitored the left ventricular pressures and contraction- and relaxation rates at baseline as well as after ischemia and reperfusion. Interestingly, the CTGF-TG and WT hearts displayed similar intracardiac pressures under basal conditions as well as under ischemia and reperfusion (supplemental Figure S3A and S3B). Similar results were obtained in respect to contraction and relaxation rates (supplemental Figure S3C and S3D). All together, these data suggest, that CTGF overexpression does not play a role in the immediate physiological response of the heart to ischemia. In addition, the acute recovery process is not affected by CTGF overexpression.

## Discussion

We produced transgenic mice and rats with cardiomyocyte-restricted overexpression of CTGF and found that CTGF alone does not induce cardiac fibrosis, but rather exerts a prohypertrophic function. Transgenic mice and rats abundantly expressed the CTGF protein in the interstitial space of the right and left ventricles. The secretion of CTGF from cardiomyocytes to the interstitium is in accordance with the localization of CTGF observed in hearts from spontaneously hypertensive rats, another model with significant CTGF upregulation. These findings confirm the physiological distribution of transgenic CTGF and support the suitability of our models for the analysis of CTGF function in the heart. Moreover, we observed in CTGF-TG mouse hearts an increased expression of the endogenous mRNA, which points to an autocrine enhancement of the CTGF regulation. This finding mirrors data published by Yokoi and colleagues, demonstrating an increased endogenous CTGF expression in kidney overexpressing transgenic CTGF [15]. Furthermore, CTGF administration also stimulates CTGF expression in cultured mesangial cells [25].

CTGF is felt to be one of the key profibrotic factors mediating the TGF-β profibrotic action. Surprisingly, the overexpression of CTGF in cardiomyocytes did not result in increased development of cardiac fibrosis neither under basal nor under hypertrophic conditions. We carefully assayed the development of fibrosis in both transgenic models by use of various methods. Our morphological observations were confirmed by collagen 1α, collagen 3, and fibronectin mRNA quantification, which also did not reveal significant changes between WT and transgenic hearts. These findings are in line with results obtained from CTGF overexpression in kidney, which also did not result in renal fibrosis under basal conditions [15]. Even after induction of diabetes in this model, no changes in the expression level of key ECM inducers like fibronectin, collagen 4A1 and 4A3 were detected. Benniaud and colleagues transiently overexpressed CTGF in lung using the adenoviral gene transfer [26]. In their study, a drastic CTGF overexpression induced a moderate pulmonary fibrosis suggesting CTGF as an indirect profibrotic factor. The cell type overexpressing CTGF may play a crucial role in the observed phenotype. Despite cardiomyocyte specific CTGF overexpression, we believe, that secretion of the protein allows it to exert its physiological function also on cardiac fibroblasts. It would be certainly intriguing to overexpress CTGF specifically in cardiac fibroblasts; however, a specific promoter for this cell type is not available until now. Interestingly, Mori at al. tested the fibrotic response of CTGF-null embryonic fibroblasts upon TGF-β stimulation. This study revealed CTGF to be dispensable for the TGF-β induced fibrotic response in embryonic fibroblasts [27]. In conclusion, our results and other studies support the hypothesis, that CTGF itself is not a fibrosis inducer. However, the protein may play a role as a profibrotic cofactor under certain conditions.

Given the possibility that CTGF is involved in contractile function and hypertrophy, we examined CTGF-TG mice by echocardiography. We observed an age dependent presence of cardiac hypertrophy, which was followed by ventricular dilatation at the age of 7 months. This phenotype was accompanied by an increased activation of the prohypertrophic Akt Kinase but surprisingly not by an increase of ANP and BNP expression. These findings are consistent with data published by Hyata et al., who showed hypertrophic effects of CTGF stimulation on cardiomyocytes *in vitro* [21]. The authors also demonstrated that hypertrophic changes of cultured cardiomyocytes were accompanied by upregulation of skeletal actin and BNP when induced by endothelin-1, but not after stimulation with CTGF. Furthermore, the study showed, that Akt activation is required for CTGF-mediated induction of hypertrophy. Interestingly, recent studies have demonstrated that Akt plays an important role in the determination of cardiac myocytes size [28]. Recently, transient activation of Akt in the heart was reported to result in adaptive/physiological hypertrophy while longer periods of Akt activation were detrimental and led to dilated cardiomyopathy [28]. These observations provide a possible explanation for the disease course observed in our CTGF-TG model. While the initial overexpression of CTGF activates Akt triggering adaptive hypertrophy, the prolonged CTGF overexpression tips the balance towards heart failure.

If overexpression of CTGF initially enhances cardiac adaptive response, the CTGF overexpressing hearts should display improved remodeling in acute cardiomyopathy. To test this hypothesis, we employed infusion of Ang II for 14 days as a model of acute pressure overload. In fact, echocardiography revealed a strong decrease of systolic function in WT mice, while CTGF-TG hearts showed preserved cardiac function that was similar to that of sham-treated animals. These results suggest a role of CTGF in the induction of cardiac adaptive response and its function as a protective factor in acute cardiomyopathy. An interpretation of these results is that CTGF plays a role as an early prohypertrophic factor that mildens or delays heart failure by reducing transmural wall tension. Such an adaptive hypertrophic response would transiently improve cardiac performance. However, in the course of progressive heart failure, the continuous expression of CTGF becomes enhanced through autocrine and paracrine regulation triggering an increased activation of prohypertrophic pathways like the Akt kinase pathway. Thus, a certain basal CTGF expression might have a beneficial effect on cardiac performance while long-term CTGF upregulation leads to “overstimulation” and activation of pathological pathways resulting in loss of cardiomyocytes and ventricular dilatation.

The altered contractile performance of CTGF-TG hearts may also be attributed to a modified Ca^2+^ handling in cardiomyocytes. To get a comprehensive picture of the cellular mechanisms accompanying the phenotypical changes in CTGF overexpressing hearts, we analyzed the Ca^2+^ metabolism and cardiomyocyte mechanisms at single cell level. Myocyte contraction and relaxation are under the control of the rise and decline in cytosolic Ca^2+^ levels [29]. The rise in intracellular free Ca^2+^ initiates contraction through the binding of Ca^2+^ to myofilaments, whereas myocyte relaxation is promoted by decrease in intracellular Ca^2+^ and dissociation of Ca^2+^ from the myofilaments. Although the cellular contractility at age of 3 months did not differ between the CTGF-TG and WT cardiomyocytes, we observed an increased reuptake of Ca^2+^ from the cytosol in CTGF-TG myocytes. A consequence of an increased Ca^2+^ reuptake might be an enhanced contractile function. However, we suggest that the observed changes in Ca^2+^ metabolism may promote increased contractility under stress conditions like upon Ang II stimulation. Lowering in cytosolic Ca^2+^ is accomplished mainly by Ca^2+^ sequestration into the sarcoplasmatic reticulum by SERCA2a and Ca^2+^ extrusion from the cell by the Na^+^/Ca^2+^-Exchanger [30]. To elucidate the contribution of both pathways to the increased Ca^2+^ reuptake in CTGF overexpressing cardiomyocytes, further experiments need to be done.

Several studies implicate CTGF as a factor being involved in tissue remodeling after myocardial infarction, but its physiological function in ischemia and following muscle recovery remains elusive [23], [24], [31]. Therefore, the present study also focused on this functional aspect of CTGF. In the working heart model we observed a similar reduction of contractility under ischemic conditions in CTGF-TG and WT hearts. Moreover, the recovery of contractile function after reperfusion was comparable in both groups. These results suggest that CTGF does not play an immediate physiological role in acute ischemic and postischemic events. We rather assume that CTGF exerts its function in later postischemic phase, where its expression is strongly induced. Dean et al. reported on major increases in CTGF mRNA and protein occurring in the viable myocardium at 180 days after myocardial infarction [31].

In summary, our current study presents evidence that CTGF itself does not induce fibrosis, but rather is a protein involved in adaptive cardiac mechanisms like cellular hypertrophy and Ca^2+^ metabolism modification. Although CTGF is broadly accepted as a profibrotic factor, there are no *in vivo* studies showing a clear causality between CTGF overexpression and fibrosis induction. So far, the major evidence for the profibrotic action of CTGF is based on the fact, that fibrotic events are accompanied by CTGF upregulation. Thus, we believe that our data provide good reason to reconsider the profibrotic role of CTGF particularly in the heart shedding new light on the functional diversity of this protein. However, detailed mechanisms of CTGF action will require further studies.

## Materials and Methods

### Generation of CTGF-transgenic mice (CTGF-TG)

We generated cardiac-directed transgenic rat (Sprague-Dawley) and mouse (FVB/N).

The full-length rat CTGF cDNA was amplified with following primers (5′-cggaattcgctgtgcgtcctcctgccg-3′ and 5′-cgggatccgagttcgtgtcccttactcc-3′) and cloned into a vector containing the mouse myosin light chain-2 promoter (MLC-2) and a region of the rabbit β-globin gene containing exon 2, intron 2 and exon 3. The construct was linearized and microinjected into the male pronucleus of mouse and rat zygotes as described [32]. The identification of founder animals and further genotyping were conducted by PCR using the primers 5′-atgttatatggagggggcaaagtt-3′ and 5′-tccacgccagccagaat-3′. Animals were kept in the hemizygote state. All animals used in this study were males. All experimental protocols were performed in accordance with the guidelines for the humane use of laboratory animals by the Max-Delbrück Center for Molecular Medicine and were approved by local German authorities with standards corresponding to those prescribed by the American Physiological Society.

### Echocardiography and Angiotensin II (Ang II) infusion

A two-dimensional short axis view of the left ventricle was obtained with a 45-MHz transducer (Sonic Vevo 770 High-Resolution Imaging System, Toronto, Canada) from Isofluran anesthetized mice. M-mode tracings were recorded and used to determine the diameter of the left ventricle at the end of the diastole (LVEDD) and systole (LVESD). Fractional shortening (FS) corresponds to the value of (LVEDD-LVESD)/LVEDD. In 3.5-months-old mice we infused Ang II (1.4 mg/kg/day) for 14 days by Alzet osmotic pumps (Alzet, Cupertino, USA) implanted subcutaneously. All animal studies were carried out in accordance with the local authorities and conforming to the National Institutes of Health Guide for the Care and Use of Laboratory Animals.

### Analysis of mRNA Expression

Total RNA was extracted from organs by using TRIzol reagent according to the manufacturer's instructions (Invitrogen, Carlsbad, USA). An antisense probe for CTGF was ^32^P-UTP-labeled by *in vitro* transcription (Riboprobe combination system Sp6/T7; Promega, Madison, USA). RNA expression was measured by RNase protection assay (RPA II kit, Ambion, Austin, USA). Protected fragments were separated on a polyacrylamide gel and detected with a Fuji phospho-imager (Fujix BAS 2000, Fuji, Tokyo, Japan). The quantification of ANP, BNP, collagen 1α, collagen 3, fibronectin, and GAPDH was carried out by Taqman-PCR. The total RNA was reverse-transcribed with oligo(dT) primers (Gibco-BRL, Carlsbad, USA) and Omniscript reverse transcriptase (Qiagen, Hilden, Germany). Primers and probes were designed using Primer Express 2.0 (Applied Biosystems, Foster City, USA). Expression levels of all genes were normalized to GAPDH RNA expression by using the 2^(−ΔΔCT)^ method. To compare samples the ΔCt-mean of the wild type group was used as a calibration sample.

### Histological Analysis

Hearts from 4- or 7-months-old CTGF-TG or control animals were perfused with 4% paraformaldehyde and embedded in paraffin. Longitudinal sections (10 µm) were stained with Masson's trichrome and Sirius red. For high magnification pictures heart tissue was perfused with 4% paraformaldehyde and postfixed with 2.5% glutaraldehyde in PBS for 48 hours. Samples were stained with 1% OsO_4_ for 2 h, dehydrated in a graded ethanol series and propylene oxide and embedded in Poly/Bed^R^ 812 (Polysciences, Inc., Eppelheim, Germany). Semithin sections (1 µm) from 7 months old animals were stained with toluidine blue. For CTGF-specific staining, hearts were cryo-preserved, cut in longitudinal sections (8 µm) and mounted onto SuperFrost Plus slides (Menzel, Braunschweig, Germany). The sections were fixed with cold acetone, washed with TBS, and incubated for 30 minutes in a humid chamber at room temperature with 10% normal donkey serum (Jackson ImmunoResearch, West Grove, USA) and washed again with TBS. Subsequently sections were incubated for 60 minutes with polyclonal anti-CTGF primary antibody (1∶500; Santa Cruz, Santa Cruz, USA). The Cy3-conjugated anti-goat IgG was uses as secondary antibody (1∶500; Jackson ImmunoResearch, West Grove, USA). Fluorescence images were collected using Axioplan2 imaging microscope and a Sensicam 12BIT camera (Zeiss, Jena, Germany).

### Western Blot

Hearts from 7-months-old CTGF-TG and control mice were homogenized in RIPA-protein extraction buffer and denatured in SDS-loading buffer (Roth, Karlsruhe, Germany) at 95°C for 5 minutes. SDS-PAGE was carried out and blotted on PVDF-membranes (Amersham, Little Chalfon, UK). Membranes were blocked with 5% (w/v) dried milk in 100 mmol/L Tris, pH 7.5, 0.1% (v/v) Tween 20 and 150 mmol/L NaCl (TBST) for 1 h prior to overnight incubation at 4°C with anti-CTGF, anti-phospho-Akt or anti-phospho-JNK (Cell Signaling, Danvers, USA) antibody as a first antibody. To quantify the extend of Akt phosphorylation and CTGF expression, the membrane was reprobed with anti-Akt antibody (Cell Signaling, Danvers, USA) or anti-GAPDH, respectively. The band intensities of Akt-phospho-protein were normalized with those of total Akt-protein. The level of JNK phosphorylation was normalized for band intensities of unspecific bands. The expression of CTGF was normalized with GAPDH expression.

### Working heart model

Three months old mice were anaesthetized with 4% chloralhydrate (Sigma Aldrich, Taufkirchen, Germany) and injected with 5,000 U/kg heparin. After thoracotomy, hearts were quickly removed, the aorta cannulated, and connected to the perfusion apparatus (Hugo Sachs electronic, March-Hugstetten, Germany). They were perfused retrogradally with Krebs-Henseleit solution (KH; mM concentrations: 118 NaCl, 4.7 KCl, 2.5 CaCl_2_, 1.2 MgSo_4_, 1.2 KH2PO_4_, 0.5 EDTA, 25 NaHCO_3_, 5.5 glucose, pH 7.4, 37°C) with a constant pressure of 50 mmHg (Langendorff mode). A Millar tip catheter was inserted into the left ventricle for measurements of pressure development and its derivatives. A small catheter was placed into the left atrium through the pulmonary vein for perfusion of the left atrium with KH at constant pressure of 10–11 mmHg (flow around 5 mL/min; preload). Registration was than switched from retrograde to anterograde perfusion (working heart mode). The perfusate exited the left ventricle through the aortic cannula, which was connected to a pressure chamber with an air cushion for the windkessel function. Aortic pressure was set to 50 mmHg (afterload). The heart was electrically paced with a constant frequency of 320 beats/min and was subjected to 40 minutes baseline perfusion, 40 minutes ischemia followed by 60 minutes reperfusion. Recordings were performed in the last 7 minutes of each phase. Signals were recorded, stored, and evaluated using software HEM (version 3.2, Notocord System).

### Isolation of adult primary cardiomyocytes

Male wild-type or CTGF-TG mice (3-months-old) were anaesthetized with isoflurane followed by intraperitoneal injection of 8 µg xylazine and 35 µg ketamine. Hearts were rapidly removed, transferred into 10 ml 0.9% NaCl solution containing 1,000 U heparin, and connected to a cannula in a Langendorff perfusion system. Hearts were perfused at 37°C for 3 minutes with Ca^2+^-free Krebs-Henseleit buffer (KHB: 127 mM NaCl, 4.6 KCl, 1.2 KH_2_PO_4_, 24.8 NaHCO_3_, 1.1 MgSO_4_, 8.3 glucose, 10 butanedione monoxime, pH 7.4, 37°C) gassed with carbogen. After that, perfusion was switched to recirculation with KH containing 0.04% collagenase (Worthington Biochemical Corporation, Lakewood, USA), and 0.2% bovine serum albumin. After 27 minutes, the hearts were pale and soft. The ventricles were minced and incubated in the digestion medium (0,23% BSA, 0.04% collagenase in KH solution) for another 10 minutes at 37°C. After filtration through a nylon mesh (200 µm pore size) and centrifugation, cells were resuspended in Ca^2+^-free medium. Ca^2+^ concentration was increased stepwise to 500 µmol/L in order to obtain Ca^2+^-tolerant cardiomyocytes. After final washes, cardiomyocytes were resuspended in M199 medium supplemented with 0.2% bovine serum albumin, 5% fetal calf serum, 5 mmol/L creatine, 5 mmol/L taurine, 2 mmol/L carnitine, 10 µmol/L cytosine-D-arabinofuranoside, and antibiotics. Cardiomyocytes were seeded in laminin-coated 4-well chamber slides (Nunc, Wiesbaden-Schierstein, Germany) specialized for microscopic contractility and fluorescence measurement, and cultured for 4 hours in M199 medium.

### Measuring cell shortening and Ca^2+^ transients

Attached cardiomyocytes were washed with Hank's balanced salts solution buffered with 10 mM Hepes at pH 7.4 (HBSS). Cells were loaded with fura-2-AM for 30 minutes at room temperature in the dark. Dye solution was removed, and cells were left on HBSS for another 15 minutes. Cell shortening and fura-signals were simultaneously measured at 30°C on an Ionoptix contractility and fluorescence system (Ionoptix, Milton, USA). Cardiomyocytes were electrically field-stimulated with bipolar pulses of 5 ms duration at 1 Hz. Cell shortening, expressed as percentage of resting cell length, was measured using the video-edge technique at a sampling rate of 240 per second. Ca^2+^ transients were monitored as ratio of fluorescence emission at 510 nm obtained by alternate excitation at 340 and 380 nm (340/380 ratio). Data files from 15 consecutive beats recorded at intervals were averaged for analysis.

### Statistical analysis

Differences between two groups were evaluated by using unpaired Student's t-test and Mann-Whitney U-test. Differences between more than two groups were evaluated by using ANOVA followed by Fisher's probable least-squares difference test using Prism software (Graphpad Software, La Jolla, USA). The significance level was set at *p*<0.05. All data are expressed as mean±SEM.

## Supporting Information

Figure S1 Fibrosis development in CTGF-TG rats and mice. (A) Expression of CTGF mRNA in heart of transgenic rats shown by ribonuclease protection assay. (B) Western Blot analyses of CTGF protein overexpression in CTGF-TG rat hearts. GAPDH protein expression was used as loading control. (C) Sirius red staining of fibrotic tissue in left ventricle of WT and CTGF-TG rat as well as CTGF-TG and WT rat treated with isoproterenol. (D) Quantification of the collagen 1α, collagen 3, and fibronectin mRNA expression by TaqMan-PCR performed in 4-months-old rats (n = 5 per group). (E) Sirius red stained cardiac sections of WT and CTGF-TG mice at age of 4 and 7 months as well as WT mice treated with isoproterenol as positive control (WT-mouse-ISO). Scale bars designate a length of 50 µm.
(13.94 MB TIF)

Figure S2 Measurements of Ca2+-cycling and contractile function in single isolated cardiomyocytes at age of 3 months. Isolated murine cardiomyocytes were measured for (A) baseline cellular length, (B) FS, (C) contraction and (D) relaxation rate. The Ca2+-cycling was assessed by recording of such parameters like (E) cytosolic Ca2+ content in diastole, (F) amplitude of the Ca2+ wave, (G) velocity of the Ca2+ influx and (H) efflux from the cytosol. CTGF-TG cardiomyocytes n = 43, WT cardiomyocytes n = 38.
(0.58 MB TIF)

Figure S3 Characterization of cardiac function at 3 months of age in ischemia/reperfusion model. The left ventricular pressures were assessed during 5 minutes of each phase (baseline, ischemia and reperfusion). We monitored (A) intracardiac pressure in systole (ICPsys), (B) intracardiac pressure in diastole (ICPdia), (C) contraction rate (ICPsys/sec), and (D) relaxation rate (ICPdia/sec), n = 7 mice per group.
(0.60 MB TIF)

## References

[1] Kannel WB (2000). Incidence and epidemiology of heart failure.. Heart Fail Rev.

[2] Levy D, Kenchaiah S, Larson MG, Benjamin EJ, Kupka MJ (2002). Long-term trends in the incidence of and survival with heart failure.. N Engl J Med.

[3] Chien KR (2000). Genomic circuits and the integrative biology of cardiac diseases.. Nature.

[4] Grotendorst GR, Okochi H, Hayashi N (1996). A novel transforming growth factor beta response element controls the expression of the connective tissue growth factor gene.. Cell Growth Differ.

[5] Holmes A, Abraham DJ, Sa S, Shiwen X, Black CM (2001). CTGF and SMADs, maintenance of scleroderma phenotype is independent of SMAD signaling.. J Biol Chem.

[6] Khan R, Sheppard R (2006). Fibrosis in heart disease: understanding the role of transforming growth factor-beta in cardiomyopathy, valvular disease and arrhythmia.. Immunology.

[7] Blom IE, Goldschmeding R, Leask A (2002). Gene regulation of connective tissue growth factor: new targets for antifibrotic therapy?. Matrix Biol.

[8] Grotendorst GR (1997). Connective tissue growth factor: a mediator of TGF-beta action on fibroblasts.. Cytokine Growth Factor Rev.

[9] Perbal B (2004). CCN proteins: multifunctional signalling regulators.. Lancet.

[10] Bork P (1993). The modular architecture of a new family of growth regulators related to connective tissue growth factor.. FEBS Lett.

[11] Roestenberg P, van Nieuwenhoven FA, Joles JA, Trischberger C, Martens PP (2006). Temporal expression profile and distribution pattern indicate a role of connective tissue growth factor (CTGF/CCN-2) in diabetic nephropathy in mice.. Am J Physiol Renal Physiol.

[12] Whitfield ML, Finlay DR, Murray JI, Troyanskaya OG, Chi JT (2003). Systemic and cell type-specific gene expression patterns in scleroderma skin.. Proc Natl Acad Sci U S A.

[13] Gauldie J (2002). Pro: Inflammatory mechanisms are a minor component of the pathogenesis of idiopathic pulmonary fibrosis.. Am J Respir Crit Care Med.

[14] Lang C, Sauter M, Szalay G, Racchi G, Grassi G (2008). Connective tissue growth factor: a crucial cytokine-mediating cardiac fibrosis in ongoing enterovirus myocarditis.. J Mol Med.

[15] Yokoi H, Mukoyama M, Mori K, Kasahara M, Suganami T (2008). Overexpression of connective tissue growth factor in podocytes worsens diabetic nephropathy in mice.. Kidney Int.

[16] Ohnishi H, Oka T, Kusachi S, Nakanishi T, Takeda K (1998). Increased expression of connective tissue growth factor in the infarct zone of experimentally induced myocardial infarction in rats.. J Mol Cell Cardiol.

[17] Chen H, Huang XN, Stewart AF, Sepulveda JL (2004). Gene expression changes associated with fibronectin-induced cardiac myocyte hypertrophy.. Physiol Genomics.

[18] Matsui Y, Sadoshima J (2004). Rapid upregulation of CTGF in cardiac myocytes by hypertrophic stimuli: implication for cardiac fibrosis and hypertrophy.. J Mol Cell Cardiol.

[19] Koitabashi N, Arai M, Kogure S, Niwano K, Watanabe A (2007). Increased connective tissue growth factor relative to brain natriuretic peptide as a determinant of myocardial fibrosis.. Hypertension.

[20] He Z, Way KJ, Arikawa E, Chou E, Opland DM (2005). Differential regulation of angiotensin II-induced expression of connective tissue growth factor by protein kinase C isoforms in the myocardium.. J Biol Chem.

[21] Hayata N, Fujio Y, Yamamoto Y, Iwakura T, Obana M (2008). Connective tissue growth factor induces cardiac hypertrophy through Akt signaling.. Biochem Biophys Res Commun.

[22] Koitabashi N, Arai M, Niwano K, Watanabe A, Endoh M (2008). Plasma connective tissue growth factor is a novel potential biomarker of cardiac dysfunction in patients with chronic heart failure.. Eur J Heart Fail.

[23] Gabrielsen A, Lawler PR, Yongzhong W, Steinbruchel D, Blagoja D (2007). Gene expression signals involved in ischemic injury, extracellular matrix composition and fibrosis defined by global mRNA profiling of the human left ventricular myocardium.. J Mol Cell Cardiol.

[24] Ahmed MS, von Lueder TG, Oie E, Kjekshus H, Attramadal H (2005). Induction of myocardial connective tissue growth factor in pacing-induced heart failure in pigs.. Acta Physiol Scand.

[25] Riser BL, Denichilo M, Cortes P, Baker C, Grondin JM (2000). Regulation of connective tissue growth factor activity in cultured rat mesangial cells and its expression in experimental diabetic glomerulosclerosis.. J Am Soc Nephrol.

[26] Bonniaud P, Margetts PJ, Kolb M, Haberberger T, Kelly M (2003). Adenoviral gene transfer of connective tissue growth factor in the lung induces transient fibrosis.. Am J Respir Crit Care Med.

[27] Mori Y, Hinchcliff M, Wu M, Warner-Blankenship M, K ML (2008). Connective tissue growth factor/CCN2-null mouse embryonic fibroblasts retain intact transforming growth factor-beta responsiveness.. Exp Cell Res.

[28] Shiojima I, Sato K, Izumiya Y, Schiekofer S, Ito M (2005). Disruption of coordinated cardiac hypertrophy and angiogenesis contributes to the transition to heart failure.. J Clin Invest.

[29] Bers DM (2002). Cardiac excitation-contraction coupling.. Nature.

[30] Piacentino V, Weber CR, Chen X, Weisser-Thomas J, Margulies KB (2003). Cellular basis of abnormal calcium transients of failing human ventricular myocytes.. Circ Res.

[31] Dean RG, Balding LC, Candido R, Burns WC, Cao Z (2005). Connective tissue growth factor and cardiac fibrosis after myocardial infarction.. J Histochem Cytochem.

[32] Re RN DD, Schiffrin EL, Sowers JR, Francis T (2006). Brain renin-angiotensin system - focus on transgenic animal models.. Molecular Mechanisms in Hypertension.

