# Correction: Connective Tissue Growth Factor Overexpression in Cardiomyocytes Promotes Cardiac Hypertrophy and Protection against Pressure Overload

**DOI:** 10.1371/annotation/818d7cc6-3ec0-4fc5-82e1-8e9b6ceca336

**Published:** 2009-09-08

**Authors:** Anna N. Panek, Maximilian G. Posch, Natalia Alenina, Santhosh K. Ghadge, Bettina Erdmann, Elena Popova, Andreas Perrot, Christian Geier, Rainer Dietz, Ingo Morano, Michael Bader, Cemil Özcelik

A comma was omitted between the names of authors nine and ten, so the two authors appear to be one. Rainer Dietz is author number nine, with affiliations 1, 2, and 3. Ingo Morano is author number ten, with affiliation number 1. The author byline and affiliations should appear as shown here:

Anna N. Panek,^1^ Maximilian G. Posch,^2,3^ Natalia Alenina,^1^ Santhosh K. Ghadge,^1^ Bettina Erdmann,^1^ Elena Popova,^1^ Andreas Perrot,^2,3^ Christian Geier,^2,3^ Rainer Dietz,^1,2,3^ Ingo Morano,^1^ Michael Bader,^1^ and Cemil Özcelik^1,2,3^

**1** Department of Cardiovascular and Metabolic Disease Research, Max Delbrück Center for Molecular Medicine, Berlin, Germany, **2** Experimental and Clinical Research Center (ECRC) at the Max Delbrück Center for Molecular Medicine, Berlin, Germany, **3** Department of Cardiology, Charité-Universitätsmedizin, Campus Virchow Klinikum, Berlin, Germany

