# Supplementary figures and images for: Connective Tissue Growth Factor Overexpression in Cardiomyocytes Promotes Cardiac Hypertrophy and Protection against Pressure Overload
Source: PLoS One. 2009 Aug 25;4(8):e6743. doi: 10.1371/journal.pone.0006743 (PMC2727794; doi:10.1371/journal.pone.0006743)

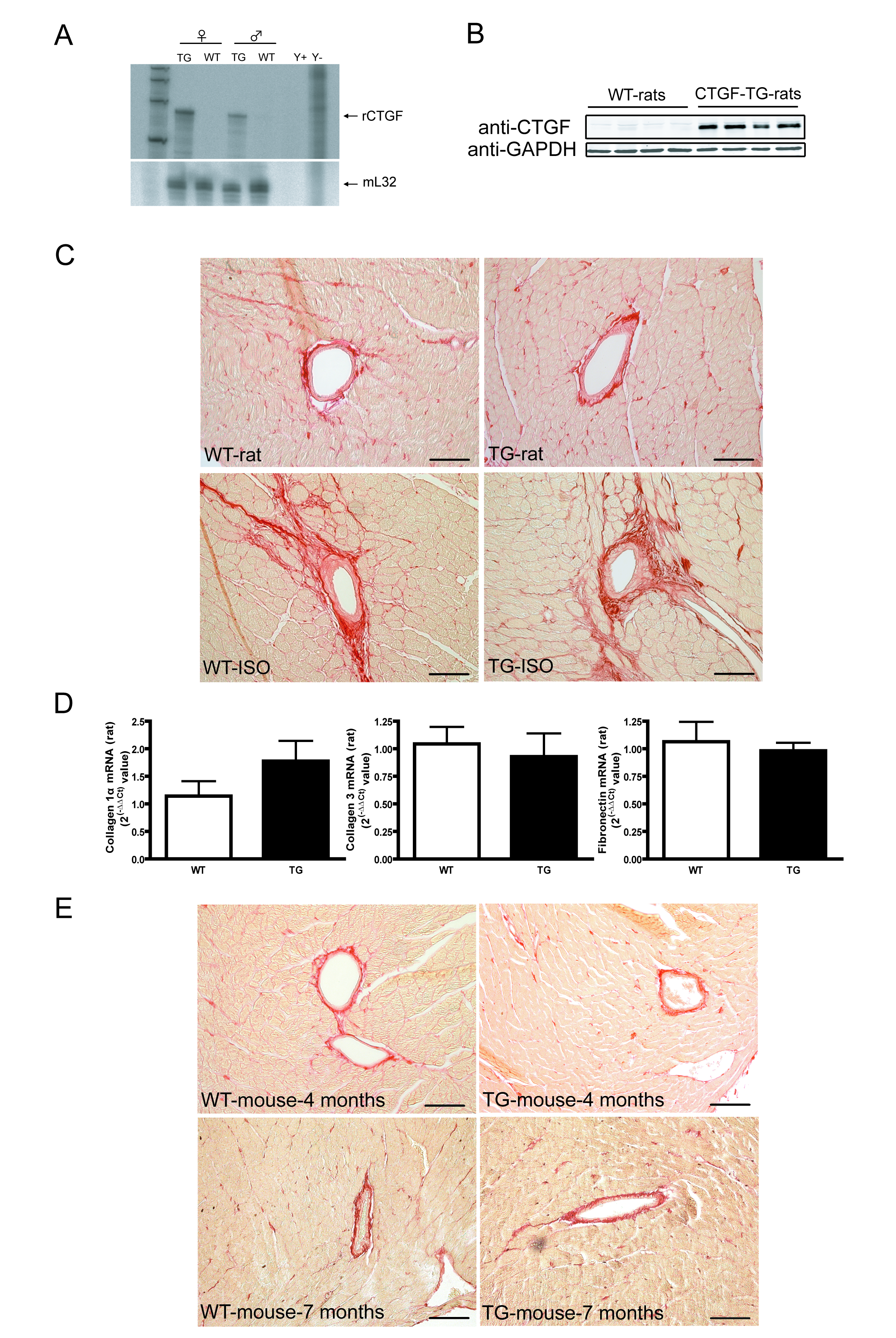

Supplement: Figure S1 — Fibrosis development in CTGF-TG rats and mice. (A) Expression of CTGF mRNA in heart of transgenic rats shown by ribonuclease protection assay. (B) Western Blot analyses of CTGF protein overexpression in CTGF-TG rat hearts. GAPDH protein expression was used as loading control. (C) Sirius red staining of fibrotic tissue in left ventricle of WT and CTGF-TG rat as well as CTGF-TG and WT rat treated with isoproterenol. (D) Quantification of the collagen 1α, collagen 3, and fibronectin mRNA expression by TaqMan-PCR performed in 4-months-old rats (n = 5 per group). (E) Sirius red stained cardiac sections of WT and CTGF-TG mice at age of 4 and 7 months as well as WT mice treated with isoproterenol as positive control (WT-mouse-ISO). Scale bars designate a length of 50 µm. (13.94 MB TIF) [file pone.0006743.s001.tif]

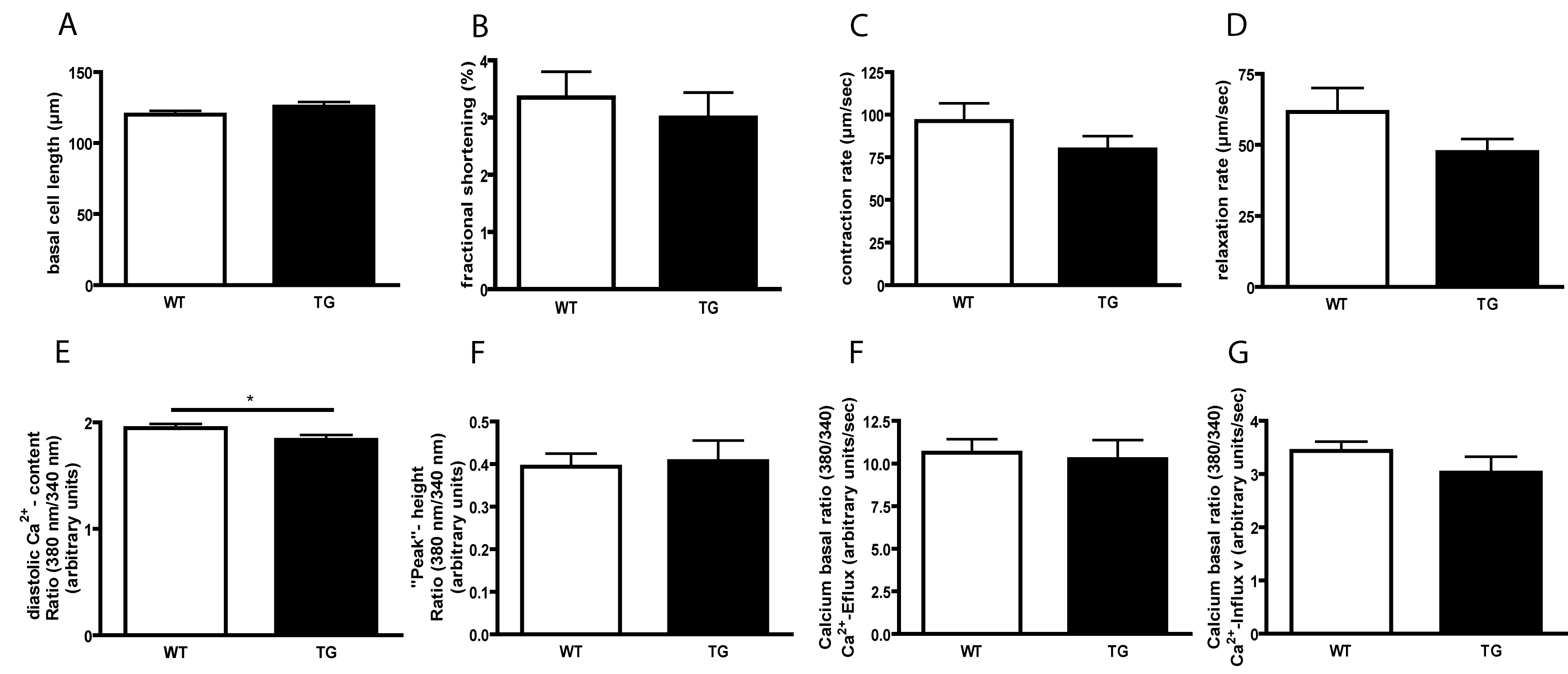

Supplement: Figure S2 — Measurements of Ca2+-cycling and contractile function in single isolated cardiomyocytes at age of 3 months. Isolated murine cardiomyocytes were measured for (A) baseline cellular length, (B) FS, (C) contraction and (D) relaxation rate. The Ca2+-cycling was assessed by recording of such parameters like (E) cytosolic Ca2+ content in diastole, (F) amplitude of the Ca2+ wave, (G) velocity of the Ca2+ influx and (H) efflux from the cytosol. CTGF-TG cardiomyocytes n = 43, WT cardiomyocytes n = 38. (0.58 MB TIF) [file pone.0006743.s002.tif]

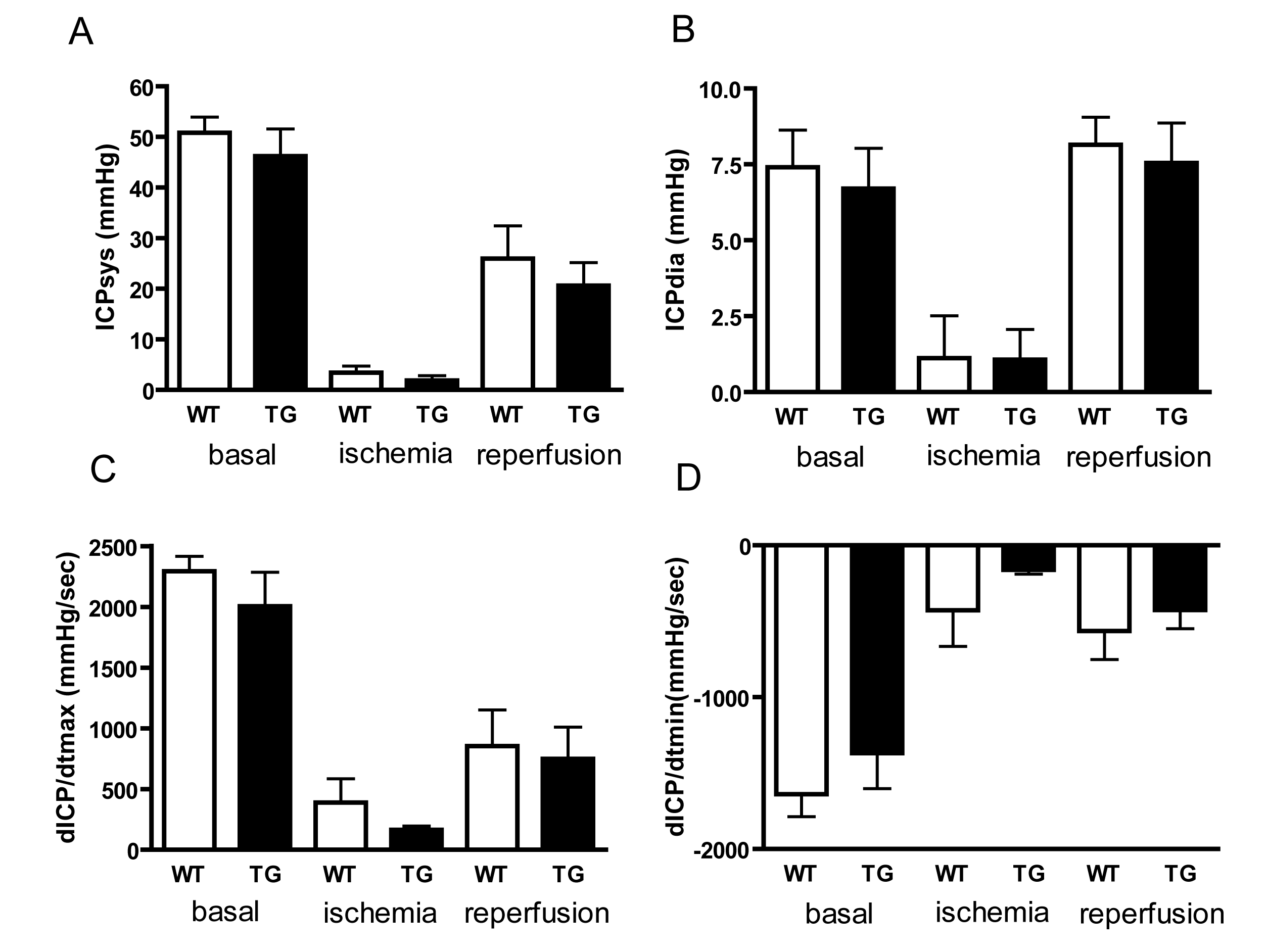

Supplement: Figure S3 — Characterization of cardiac function at 3 months of age in ischemia/reperfusion model. The left ventricular pressures were assessed during 5 minutes of each phase (baseline, ischemia and reperfusion). We monitored (A) intracardiac pressure in systole (ICPsys), (B) intracardiac pressure in diastole (ICPdia), (C) contraction rate (ICPsys/sec), and (D) relaxation rate (ICPdia/sec), n = 7 mice per group. (0.60 MB TIF) [file pone.0006743.s003.tif]
